# Value of different CTO scoring systems in predicting procedural success in coronary chronic total occlusion intervention in Egyptian patients

**DOI:** 10.1186/s43044-024-00458-6

**Published:** 2024-03-05

**Authors:** Ahmed Mohammed Ali AlAshry, Muhammed Nagy Nagiub, Magdy Farouk Ahmed Ismael, Wesam Alghonaimy

**Affiliations:** 1 Cardiology Department, Faculty of Medicine, Cairo University, Cairo, Egypt; 2 Cardiology Department, Faculty of Medicine, Helwan University, Third East District, Area 9, Villa 28, El Sherouk City, Cairo, Egypt

**Keywords:** Chronic total occlusion (CTO), Percutaneous coronary intervention (PCI), CTO scoring systems, Egyptian patients

## Abstract

**Background:**

Chronic total occlusion (CTO) lesions in coronary arteries pose a significant challenge for coronary interventionists, often leading to referrals for coronary artery bypass graft surgery (CABG). Successful percutaneous coronary intervention (PCI) for CTOs requires accurate assessment of procedural potential. This study, comprising 100 Egyptian patients aged 37–81, compares the predictive efficacy of various CTO scoring systems in PCI success determination. Patients with CTO in at least one coronary artery, planned for elective PCI based on objective evidence of ischemia, were included. Experienced operators performed PCI, recording procedural variables, and assessing complications. Logistic regression analysis revealed an inverse linear relationship between success rates and score values across all systems.

**Results:**

Although, the predictive capacity of the scores was similar, with slight differences. The Euro CTO (CASTLE) score^10^ exhibited superior predictive efficacy, followed by the CL score^9^, while PROGRESS^8^ and J-CTO^7^ scores showed lower significance. ORA CTO^11^ score demonstrated intermediate predictive ability, and PROGRESS score^8^ had the least predictive value.

**Conclusion:**

The CASTLE score^10^ proved most effective in predicting PCI success for CTO cases in Egyptian patients, with operators advised to choose scoring systems based on experience and case characteristics. Proper planning remains crucial for optimizing success rates in CTO PCI procedures, irrespective of the scoring system employed.

## Background

Chronic total occlusion (CTO) lesions remain a formidable challenge for coronary interventionists and often prompt referrals for coronary artery bypass graft surgery (CABG) [1]. Percutaneous coronary intervention (PCI) success rates for CTOs range from 55 to 80%, with specialized centers achieving higher success rates [2].

The evidence supporting CTO treatment is grounded in registry observations and recent randomized trials [3, 11]. Recent guidelines advocate a unified approach to revascularization, yet the technical intricacies of CTO PCI demand specialized management [4]. Complications such as dissection, perforation, and collateral impairment to the distal bed are associated with this procedure. Following a successful CTO PCI, the major adverse coronary event (MACE) rate is approximately 2 to 2.5% [2], while failed PCI is linked to a higher MACE rate of approximately 5.6% [1].

Successful CTO PCI has demonstrated improved clinical outcomes, including reduced mortality, angina, stroke risk, and the need for subsequent CABG [5]. Given the procedure's complexity and potential complications, meticulous patient selection [6] and planning are imperative. Advances in equipment, techniques, and scoring systems contribute to enhanced success rates and outcomes in CTO PCI procedures [6, 11].

Numerous scoring systems have been developed to assess the potential success of CTO PCI procedures. Widely utilized globally are the J CTO score (Multicenter CTO Registry in Japan) [7], the Prospective Global Registry for the Study of Chronic Total Occlusion Intervention score (PROGRESS CTO) [8], the clinical and lesion-related score (CL) by Alessandrino et al. [9], the Euro CTO (CASTLE) [10], and the ORA score (Ostial Location, Age ≥ 75 years, Rentrop Grade less than 2) by Galassi et al. [11]. Table 1 summarizes different scores definitions taken from the original publications [7–11].

**Table 1:** Comparison of diffrent variables of CTO scoring systems used in this study [15]

Criteria of Scores	J-CTO (0–5)	Progress-CTO (0–4)	Euro (CASTLE-CTO) (0–6)	CL-CTO (0–8 by 0.5)	ORA-CTO (0–4)
Calcification	Calcification Existence		Calcification Existence	Calcification Existence**	
Tortuosity	Bending > 45	Presence of Two bends > 70 degrees or 1 bend > 90 degree	Presence of two bends > 90 or 1 bend > 120		
Age			Age > 70		Age > 75
Stump	Blunt Proximal Cap	Proximal Cap Ambiguity	Blunt	Blunt	
Length	Occluded segment > 20 mm		Occluded segment > 20 mm	Occluded segment > 20 mm*	
Redo (Previous Failed Attempt)	Yes				
CABG (History)			Yes	Yes*	
MI (History)				Yes	
Location of CTO		LCX		Non-LAD	
Interventional Collaterals		Absence			Collateral Filling**
Ostial CTO					Yes

The score of each item is equal to 1 point except for * = 1.5 points and ** = 2 points

These scoring systems serve multiple purposes, providing a numerical assessment of success and complications, facilitating improved case selection based on objective anatomical and clinical complexity assessments [8]. Additionally, they guide decision-making within the heart team by customizing the revascularization approach for each patient, considering the objective probability of technical/angiographic success with PCI. Furthermore, CTO scores offer a valuable framework for reviewing coronary angiograms [12] and standardize the classification of CTO lesion complexity, enabling result comparisons across operators, facilities, countries, and regions for both clinical research and quality enhancement [8].

This study aims to compare the accuracy of different CTO Scoring Systems in Predicting the Procedural Success of Percutaneous Coronary Intervention in Egyptian Patients. J CTO^7^, PROGRESS^8^, CL^9^, CASTLE^10^, and ORA^11^ CTO scores will be calculated using coronary angiography and medical documentation, with procedural success serving as the primary endpoint.

## Methods

Prior to study participation, written informed consent was obtained from all patients following a comprehensive explanation of the procedure. The study adhered to the principles of the Declaration of Helsinki and received approval from the Ethical Committee of the Faculty of Medicine, Helwan University.

A prospective comparative study was conducted using a convenience sampling technique at the cardiology department of Helwan University Hospital (Badr Hospital) and International Medical Center of Egypt from December 2021 to February 2023. The study enrolled 100 patients with an average age range of 37 to 81 years, consisting of 94 males and 6 females. Inclusion criteria encompassed patients with chronic total occlusion (CTO) in at least one coronary artery, planned for an elective percutaneous coronary intervention (PCI) trial based on objective evidence of ischemia or persistent ischemic symptoms attributed to the target artery supplying a viable myocardial area.

The diagnostic criteria for CTO included the obstruction of a native coronary artery with no luminal continuity, presenting thrombolysis in myocardial infarction (TIMI) flow grade 0 or 1 for over 3 months, as determined through clinical information or previous angiography results. Included CTOs were classified as either angiographically confirmed (certain) or clinically confirmed (likely), with exclusion criteria comprising hemodynamically unstable patients, baseline renal impairment (serum creatinine > 1.4 mg/dl), severe left ventricular dysfunction (EF < 30%), irregular cardiac rhythm (e.g., atrial fibrillation, frequent extrasystoles), and non-viable myocardium in the territory supplied by the chronic totally occluded artery, confirmed through viability studies e.g., Dobutamine stress echocardiography.

All study patients underwent thorough preparation, including comprehensive history taking including risk factors assessment of hypertension, diabetes, hyperlipidemia and smoking [15], general and local cardiac examination, resting 12-lead electrocardiogram (ECG), transthoracic echocardiography for left ventricular (LV) systolic function assessment by calculating LV ejection fraction, and adequate pre-PCI preparation with a loading dose of 300 mg of clopidogrel or 180 mg of ticagrelor, followed by maintenance doses. Serum creatinine levels were assessed before PCI.

- Pre-PCI
  Experienced operators calculated CTO scores, including the J-CTO score, PROGRESS CTO score, Euro CTO score (CASTLE), CL CTO score, and ORA CTO score. The calculated scores considered various variables (Table 1).
- PCI
  PCI procedures were conducted by highly skilled operators specialized in CTO interventions.
- Post PCI: The following parameters were documented for all patients:
  - Procedural Success (Primary Endpoint): Defined as achieving a residual diameter stenosis < 30% and a TIMI flow rate of grade 2 or 3.
  - Approach Success: Determining success or failure of the approach, whether antegrade or retrograde.
  - Immediate Post-Procedural Complications: Events occurring within 48 h post-procedure, such as contrast-induced nephropathy and peri-procedural myocardial infarction.

All data collected from patients were comprehensively processed, analyzed, interpreted, and statistically evaluated and Logistic regression analysis was done.

Statistical analysis was conducted using SPSS 22 for Windows (SPSS Inc., Chicago, Illinois, USA). Continuous variables were presented as mean ± standard deviation and range, while incidence was expressed as percentages. Student’s t-test was employed for comparing continuous variables, and chi-square test or Fisher’s exact tests were used for categorical variables, as appropriate. A significant level of ≤ 0.05 was considered.

## Results

In this study, a total of 100 CTO patients were included, with ages ranging from 37 to 81 years (Mean age ± SD: 59.2 ± 10.14). Males constituted 96% (*n* = 96) of the participants, with females comprising 4% (*n* = 4). Among the patients, 51% (*n* = 51) had diabetes mellitus (DM), 57% (*n* = 57) had hypertension, 80% (*n* = 80) were dyslipidemic, and 76% (*n* = 76) were smokers. Notably, 10% (*n* = 10) had a history of previous coronary artery bypass grafting (CABG), 23% (*n* = 23) had undergone previous PCI, and 16% (*n* = 16) had experienced a previous myocardial infarction (MI) (Table 2).

**Table 2:** Demographic and clinical characteristics of selected population presented in percentages or mean ± SD

Variable	
Age	59.2 ± 10.14
Sex (male)	94%
DM	51%
Hypertension	57%
Dyslipidemia	80%
Smoking	76%
*Culprit Artery*	
LCX	17%
RCA	51%
LAD	30%
Ramus	2%
Osteal CTO	16%
Previous CABG	10%
Previous PCI	23%
Previous MI	16%

LAD, Left anterir descending artery; LCX, Left circumflex artery; RCA, Right coronary aretry; CTO, Chronic total occlusion; CABG, Coronary artery bypass grafting; PCI, Percutaneous coronary intervention; MI, Myocardial infarction

**Fig. 1:**
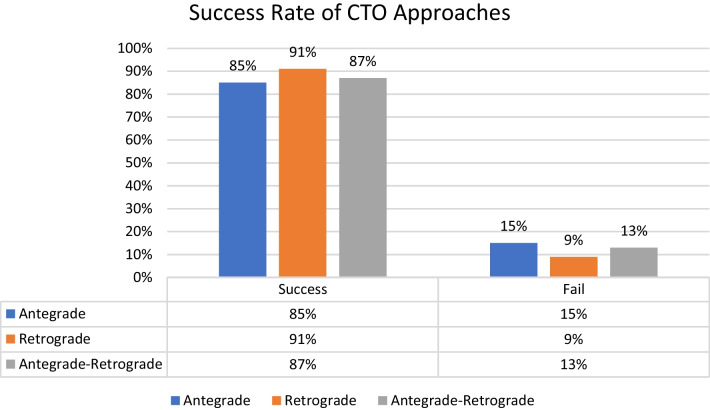
Shows percentages of success for different CTO approaches in relation to selected patients

The overall success rate for the included patients in the study was 86%. Among the selected patients, an antegrade approach was performed in 80%, with a success rate of 85%. Alternatively, a retrograde approach was undertaken in 20% of patients, yielding a higher success rate of 91% (see Fig. 1).

Patients older than 59 years old had a significantly increased incidence of failure for the whole procedure (*p* = 0.05) and Patients who suffered a previous MI had a significantly larger incidence of failure (*p* = 0.02) Compared to those who did not have previous MI.

Immediate post-procedure complications occurred in the form of Contrast-Induced Nephropathy (CIN) in 12% of the selected patients, with an overall success rate of 91% of this group of patients (those who reported CIN) compared to 9% failure rate in the same group of patients (those who reported CIN).

Post-procedure Myocardial Infarction (MI) occurred in 7% with an overall success rate of 85% of this group of patients (those who reported MI) compared to 15% failure rate in the same group of patients, and both complications occurred in 3% of the patients.

Regarding the predictive value of different CTO scoring systems for procedural outcomes, the mean and standard deviation for each scoring system were as follows: J-CTO Score was 2.47 ± 1.13; Progress CTO 1.06 ± 0.96; Euro (CASTLE) CTO 2.13 ± 1.13; CL CTO 3.98 ± 1.58; ORA CTO 1.22 ± 1.21 (Table 3).

**Table 3:** Percentages or mean ± SD of different scores

Score	Mean	Standard deviation
J-CTO	2.47	1.13
Progress-CTO	1.06	0.96
Euro (CASTLE-CTO)	2.13	1.13
CL-CTO	3.98	1.58
ORA-CTO	1.22	1.21

**Fig. 2:**
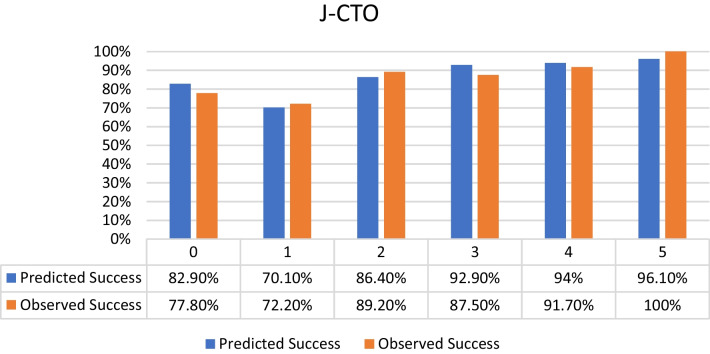
Expected success rate compared to observed success rate for J-CTO with Linear trend *p* value *p* < 0.001

**Fig. 3:**
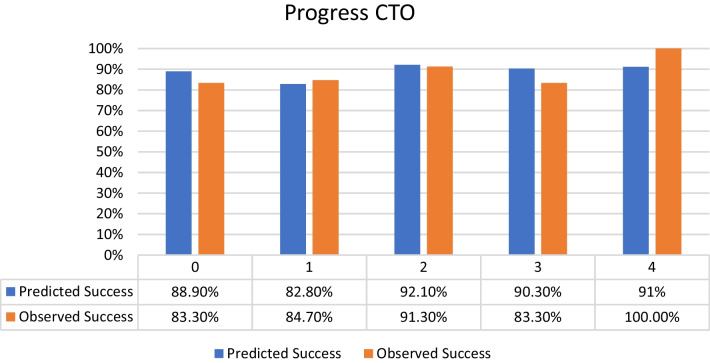
Expected success rate compared to observed success rate for Progress CTO with Linear trend *p* value *p* < 0.001

**Fig. 4:**
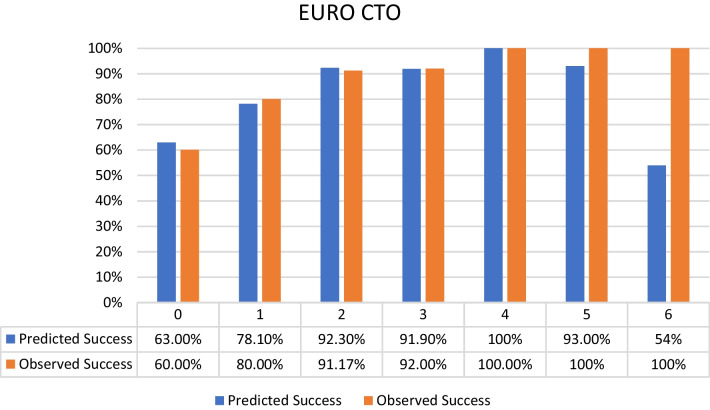
Expected success rate compared to observed success rate for Euro CTO with Linear trend *p* value *p* < 0.001

**Fig. 5:**
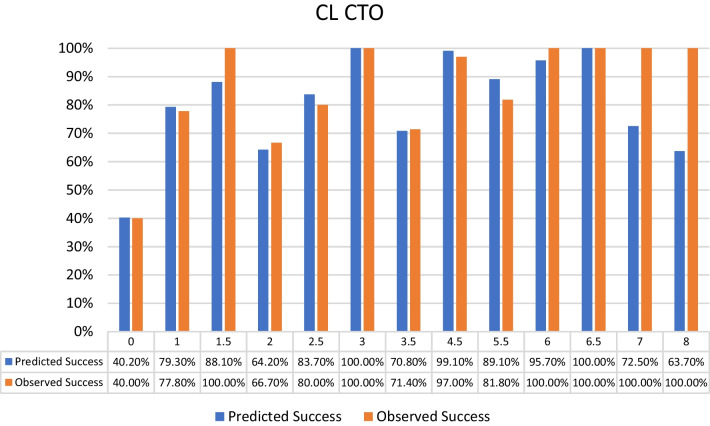
Expected success rate compared to observed success rate for CL CTO with Linear trend *p* value *p* < 0.001

**Fig. 6:**
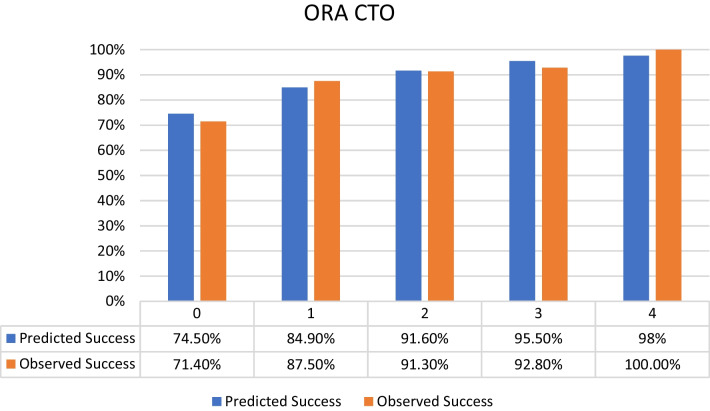
Expected success rate compared to observed success rate for ORA CTO with Linear trend *p* value *p* < 0.001

Distribution of the selected patients according to the different CTO scores was as follows:

- J-CTO score^7^ distribution: 9% score 0 (easy), 18% score 1 (intermediate), 37% score 2 (difficult), 36% score 3–5 (very difficult) from the selected population with the success percentage in each score subgroup shown in Fig. 2.
- PROGRESS CTO score^8^ distribution: 24% score 0 (easy), 46% score 1 (intermediate), 23% score 2 (difficult), 7% score 3–4 (very difficult) from the selected population with the success percentage in each score subgroup shown in Fig. 3.
- Euro (CASTLE) CTO score^9^ distribution: 33% score 0–1 (easy), 34% score 2 (intermediate), 25% score 3 (difficult), 8% score 4–6 (very difficult) from the selected population with the success percentage in each score subgroup shown in Fig. 4.
- CL CTO score^10^ distribution: 14% score 0–1 (easy), 10% score 1.5–2.5 (intermediate), 58% score 3–4.5 (difficult), 18% score 5–8 (very difficult) from the selected population with the success percentage in each score subgroup shown in Fig. 5.
- ORA CTO score^11^ distribution: 21% score 0 (easy), 40% score 1 (intermediate), 23% score 2 (difficult), 16% score 3–4 (very difficult) from the selected population with the success percentage in each score subgroup shown in Fig. 6.

Logistic regression analysis revealed an inverse linear relationship between procedural success rate and score value (*p* < 0.001 for all scores). Comparison between predicted and observed success rates for each score, based on logistic regression analysis, indicated overestimation for actual success rates in lower categories of each score and underestimation in higher categories (Figs. 2, 3, 4, 5, 6).

**Fig. 7:**
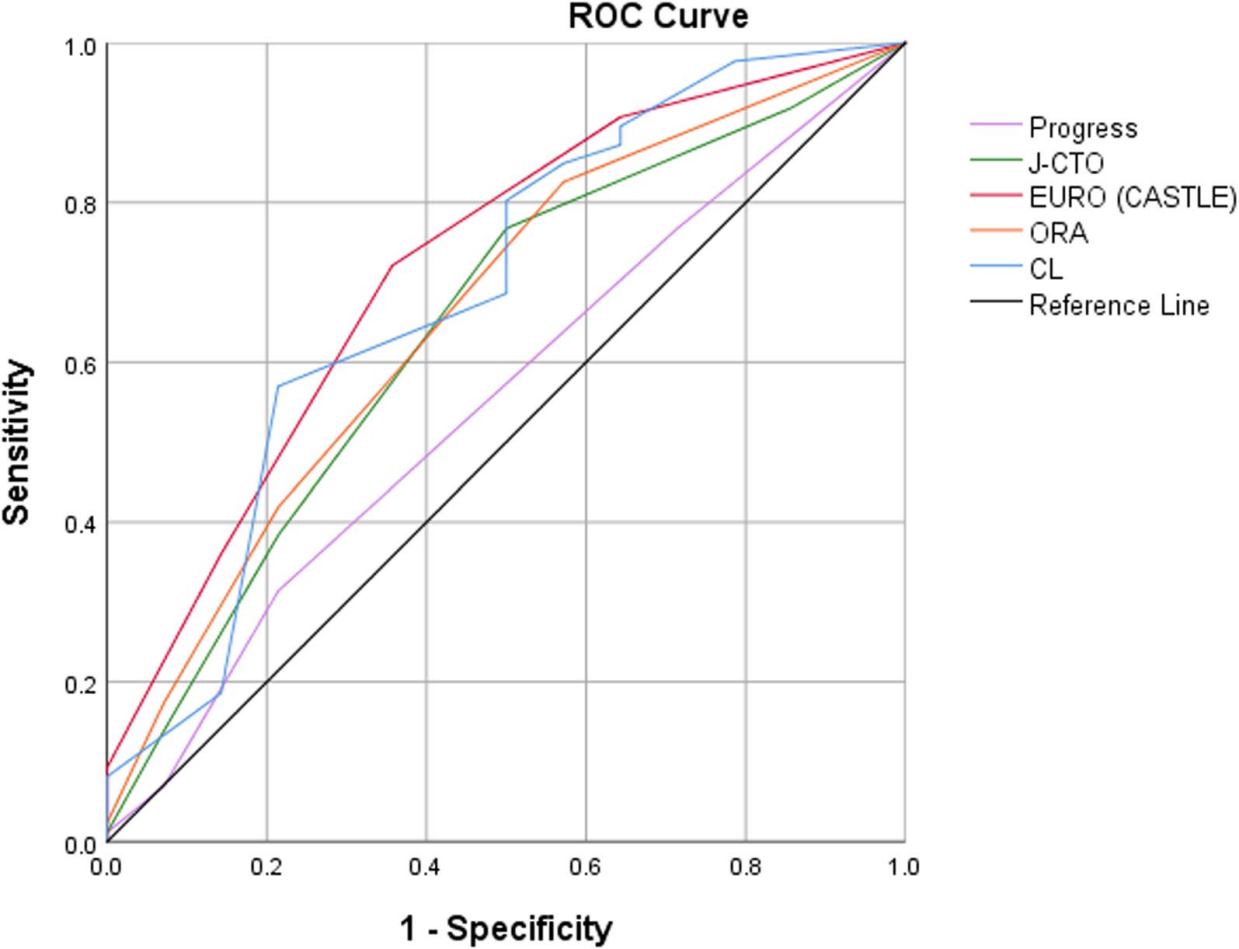
Shows ROC curve and AUC curve each score discriminating procedural success

The discrimination of the scores for procedural success was tested using the Area Under the Curve (AUC) of the Receiver Operating Characteristic (ROC) curve (see Fig. 7).

The predictive capacity of different CTO scores appeared to be near to each other with small differences, but AUC for PROGRESS score was lower than other scores (AUC 0.553, 95% CI: 0.53–0.59, *p* < 0.007), followed by J-CTO score (AUC 0.645, 95% CI: 0.62–0.68, *p* < 0.001), ORA score (AUC 0.663, 95% CI: 0.64–0.70, *p* < 0.001), CL score (AUC 0.691, 95% CI: 0.67–0.73, *p* < 0.001), and EURO (CASTLE) CTO score (AUC 0.721, 95% CI: 0.70–0.76, *p* < 0.001). This indicates that PROGRESS and J-CTO scores were less significant in predicting the success rate, while EURO (CASTLE) CTO score was superior to other scores, with small differences higher than CL score. ORA CTO score shows intermediate probability in predicting the success of PCI in CTO cases (see Table 4).

**Table 4:** Shows ROC curve and AUC curve each score discriminating procedural success

	AUC	AUC CI
Progress	0.553	0.53–0.59
J-CTO	0.645	0.62–0.68
EURO (CASTLE)	0.721	0.70–0.76
ORA	0.663	0.64–0.70
CL	0.691	0.67–0.73

## Discussion

Analysis of demographic and clinical characteristics of our study patients in relation to PCI outcome revealed a significant effect of patient’s age on the procedure outcome with more failure in patients older than 59 years old. The effect of age may be related to increased CTO duration. Additionally, increased age seems to be related to more severe calcification. In addition to that, patients who suffered from a previous MI had a significantly larger incidence of failure.

An important point was observed in this study that calculating different scores to the same patients (Not different cohorts) were important due to the presence of different variables and criteria for each score and for proper selection of the best score suitable for application on the Egyptian patients. As for the heterogeneous characteristics of different cohorts may be the direct cause for the appearance of different scores worldwide with different criteria and categories for each score except for some similarities between them.

As only Euro CASTLE^10^ and CL^9^ scores has clinical variables included in their criteria as for previous CABG for example which is a known factor that increases the failure rate in the CTO PCI with a recent study from the progress database that found 5% less recanalization success in CABG patients compared to non-CABG patients [13]. Another important note was that the definition of tortuosity and calcification were slightly different between different scores and may be depending on the operator himself to some extent, but they were still the same for severe tortuosity and severe calcification in different scores as they considered to be from the important factors to be consistently calculated. And it is notable that only two scores considered the evaluation of collateral circulation (PROGRESS^8^ and ORA^11^ scores) despite their importance in planning for the best approach in the CTO PCI. And despite these differences, it is very important to make a proper evaluation for each CTO case calculating one or more CTO scores for proper classification of the case complexity and for procedural planning especially for operators early in the CTO PCI learning curve.

Comparing the predictive results of the success incidence of different CTO scores in our study with the original values of the different cohorts of the different scores it was higher at our study this may be due to the new devices used in the current study and techniques used nowadays performing more complex cases in comparison to the older J-CTO^7^, Progress^8^ and CL^9^ scores in contrary to the Euro CASTLE^10^ CTO score which is the most recent with derived data from Euro CTO club.

There have been some published studies of score comparisons suggested that the scores may perform as well or have a better prediction than the original cohorts of different scores. Karastakis et al. compared J-CTO^7^ and PROGRESS^8^ scores in a cohort from the PROGRESS CTO registry [14] which reported that The PROGRESS CTO score is a simple tool that can be used in clinical practice to predict CTO PCI success and guide clinical decision-making.

Meanwhile, another comparison was made between J-CTO^7^, PROGRESS^8^ and CL^9^ scores to CASTLE^10^ score which showed the same results of the original cohorts Salinas et al. [15].

In our study PROGRESS^8^ and J-CTO^7^ score were less significant in prediction of success rate while EURO (CASTLE)^10^ CTO score was superior to other scores with small differences higher than CL^9^ score. ORA^11^ CTO score shows intermediate probability in prediction of success of PCI in CTO cases which is the same results of Salinas et al. [15] with nearly the same order of the used CTO scores in both studies except for the ORA score which is not used in the other study of Salinas et al. [15] as they only compared J-CTO^7^, PROGRESS^8^, CL^9^ and EURO (CASTLE)^10^ CTO scores.

An interesting observation was noted which is that the observed success rates were higher than predicted success rates in the higher categories of different scores and on the other hand the predicted success rates were higher than the observed ones in the lower categories of different scores. That means that there was an overestimation for the actual success rates in the lower categories of each score and an underestimation in the higher categories. This could also means there should be a proper planning for each CTO case with calculating of at least two CTO scores for reaching a better success result and this should be done especially for less experienced operators unlike very experienced operators with higher success rates (over 90%) will be less interested in calculating CTO scores in the purpose of knowing success rate but in order to know the risk of different complications especially for CASTLE^10^ and CL^9^ score respectively (Both have Clinical variables in addition to the angiographical ones, Table 1) so both scores may be used for the prediction of the risk of complications. Although CASTLE is easier to calculate as it has less criteria than CL score.

Finally, and according to our study CASTLE^10^ score is the best score in predicting the success of PCI in CTO cases among the Egyptian patients assessed in this study as it was originally depending on the largest cohort, operators, and techniques between the other scores, and it contains clinical variables and less criteria to be counted Table 1.

## Conclusions

According to this study, it was found that CASTLE^10^ score was the best predictive score to be calculated for the prediction of success among Egyptian patients who participated in this study.

This was for multiple reasons as CASTLE^10^ score was originally depending on the largest cohort, operators and techniques between the other scores and it contains clinical variables and less criteria to be counted, followed by CL^9^ score with slight differences in between but more criteria in CL^9^ score (Table 1).

CASTLE^10^ and CL^9^ scores were followed with ORA^11^ CTO score which showed intermediate predictive value followed by J-CTO^7^ and progress^8^ scores with slightly lower predictive value.

It could be suggested that different operators should choose the proper CTO score according to their experiences either for the proper choice of the CTO cases to perform or for minimizing the risk of complications and according to our study CASTLE^10^ and CL^9^ scores were the best predictors of success of CTO PCI for both experienced and less experienced operators.

Many factors influence the difficulty of the CTO PCI not only the individual criteria of different scores but also the criteria of the cohort included.

Finally, there should always be a proper planning for each CTO case whatever the CTO score is to guarantee the maximum chances of success.

## Limitations

There are some limitations in this study including small sample size of recruited patients, short follow up time and Other CTO scores that were beyond the scope of this study due to the presence of special technique or device (Cross-Boss and hybrid techniques in Europe, RECHARGE registry [16]; or the use of other methods for the assessment of the CTO cases (CT-RECTOR [17] or KCCT [18] scores) so they were excluded from this study depending only on the more commonly and widespread scores.

## Data Availability

The datasets used and/or analyzed during the current study are available from the corresponding author on reasonable request as all data generated or analyzed during this study are included in this article.

## Abbreviations

ACS: Acute coronary syndrome
ACT: Activated clotting time
BMI: Body mass index
BMS: Bare metal stent
CA: Coronary angiography
CABG: Coronary artery bypass grafting
CACS: Coronary artery calcium score
CAD: Coronary artery disease
CART: Combined antegrade and retrograde subintimal tracking
CC: Collateral connection
CTO: Chronic total occlusion
DES: Drug eluting stent
DM: Diabetes mellitus
ECG: Electrocardiogram
HR: Heart rate
HTN: Hypertension
IU: International unit
IV: Intravenous
IVUS: Intravascular ultrasound
JNC: Joint National Committee on Prevention, Detection, Evaluation, and Treatment of High Blood Pressure
Kv: Kilovolt
LAD: Left anterior descending coronary
LM: Left main coronary
LV: Left ventricle
LVEF: Left ventricular ejection fraction
MACE: Major adverse cardiac events
Mg: Milligram
Mm: Millimeter
MRCD: Maximum recommended contrast dose
MRI: Magnetic resonance imaging
Ms: Millisecond
MSCT: Multislice CT
OTW: Over the wire
PCI: Percutaneous coronary intervention
PE: Pulmonary embolism
PET: Positron emission tomography
PTCA: Percutaneous transluminal coronary angioplasty
RCA: Right coronary artery
TIMI: Thrombolysis in myocardial infarction
